# Influence of visual cues on oviposition site searching and learning behavior in the parasitic beetle *Dastarcus helophoroides* (Fairmaire) (Coleoptera: Bothrideridae)

**DOI:** 10.1038/s41598-018-35580-4

**Published:** 2018-11-26

**Authors:** Fei Lyu, Xiao-xia Hai, Zhi-gang Wang, Yongguo Bi

**Affiliations:** Key Laboratories for Germplasm Resources of Forest Trees and Forest Protection of Hebei Province, College of Forestry, Agricultural University of Hebei, Baoding, Hebei China

## Abstract

Color cues play a key role in the location of hosts and host habitats; learning behavior can allow parasitoids to explore different hosts and reduce environmental uncertainty. However, it remains unclear whether the parasitic beetle *Dastarcus helophoroides* (Fairmaire) uses and learns visual cues to locate oviposition sites. In this study, we investigated the ability of females to respond to colors and associate the presence of a simulated oviposition site—wood with a trough—with colored substrates after training. Two sets of experiments were conducted: (i) investigating the innate preference for substrate coloration and (ii) investigating the ability to learn to associate substrate color with the presence of simulated oviposition sites, with the beetles being trained to respond to different substrate colors with simulated oviposition sites in sessions on 10 consecutive days. The parasitic beetles displayed an innate preference for the black substrate, but this preference changed after the beetles were trained on substrates of different colors. In the associative learning tests, these beetles laid more eggs on the reward-conditioned substrates than on the black substrate after being trained. Our results suggest that visual cues are learned and used by *D. helophoroides* during their search for and selection of oviposition sites.

## Introduction

Host foraging by parasitic insects, including parasitoid wasps and parasitic beetles, can be considered a form of habitat selection^1^. This process is often viewed as a step-by-step searching process, in which female parasitoids must first locate plants inhabited by their hosts, select those that present a high probability of finding a host, and finally search within this microhabitat for the host^2–5^. Parasitic insects use olfactory, visual, vibrational, tactile, and acoustic cues from plants, hosts, or host frass to recognize host habitats^2,6–8^. Chemical cues play major roles in the host-searching behavior of parasitic insects, and a great deal of research has been devoted to this subject in recent years^9,10^. However, although *Dastarcus helophoroides* (Fairmaire) (Coleoptera: Bothrideridae) is an important parasitoid of many longhorn beetles, there is relatively little information related to the use of visual cues by this species during host and host habitat searching.

The longhorn beetles comprise more than 35,000 species in approximately 4,000 genera. Many species are important pests of forest, plantation, orchard and urban trees and may act as vectors of tree diseases^11^. These beetles cause heavy economic losses in forests around the world, as their larvae typically burrow in the tissues of woody plants^11^. This burrowing behavior limits the effectiveness of chemical pesticides in controlling the larvae. Therefore, biological control using existing natural enemies is an important research direction. The parasitic beetle *D. helophoroides* is an important parasitoid of several longhorn beetle species in its native range, including *Anoplophora glabripennis* Motschulsky, *Monochamus alternatus* (Hope), *Massicus raddei* Blessig and *Batocera horsfieldi* (Hope)^12–14^. *D. helophoroides* is distributed widely in Kyushu and Osaka, Japan^15^; Korea^16^; and China^17^. Females deposit egg clusters on the outer surface of the bark near a host entrance or frass extrusion hole or on larval tunnel walls, and the hatched larvae search for hosts and paralyze them^18^. This behavior raises interesting questions related to the host habitat and host detection by *D. helophoroides*, including the following fundamental question: Do adult *D. helophoroides* use olfactory and visual cues from the host and host habitat to locate suitable oviposition sites? Olfactory cues from frass and tunnels produced in wood by longhorn beetle larvae have been identified; these cues are used by *D. helophoroides* to identify host habitats and find hosts within them^6,19,20^. However, nothing is known about the visual cues involved in the identification of hosts and host habitats by *D. helophoroides* adults.

*Dastarcus helophoroides* is classified as a generalist or polyphagous parasitoid because it can parasitize the larvae of many longhorn beetle species^6,12^, which typically burrow in the living or dead tissues of woody plants in various habitats^11^. Generalist species must typically contend with a larger variety of cues than must specialists during host and host habitat selection^2^. Stephens^21^ and Aquino *et al*.^7^ suggested that if a host or its habitat is variable, the evolution of learning behaviors can allow parasitoids to explore different hosts and reduce environmental uncertainty. Generalist parasitoids can retain information to allow them to distinguish novel hosts and host microhabitats by associative learning. For example, visual cues (color and shape) are acquired by female *Diachasmimorpha longicaudata* (Ashmead) through associative learning and play an important role in their search for host habitats^2^. However, the visual cues were used and learned by female *D. helophoroides* to search for hosts and host microhabitats and identify appropriate oviposition sites have rarely been addressed in previous research.

As female *D. helophoroides* deposit egg clusters near the host entrance or frass extrusion hole in natural environments^18^, they may use the color of the bark near the host entrance or frass extrusion hole and the visual and olfactory cues from larval frass to locate hosts and host habitats. The larval host entrance and frass extrusion holes are created in bark of different colors, and the bark also varies in color according to the species of plant inhabited by the host larvae. Many researchers have reported that female parasitoids can learn to differentiate the visual or olfactory cues associated with oviposition sites^3^. Therefore, the aims of this study were to investigate whether *D. helophoroides* females use visual cues (color substrate) to locate oviposition sites and able to locate and identify special color substrates during the search for oviposition sites, as well as to demonstrate their ability to associate the presence of simulated oviposition sites with substrate color through associative learning.

## Results

### Innate preference

**Figure 1:**
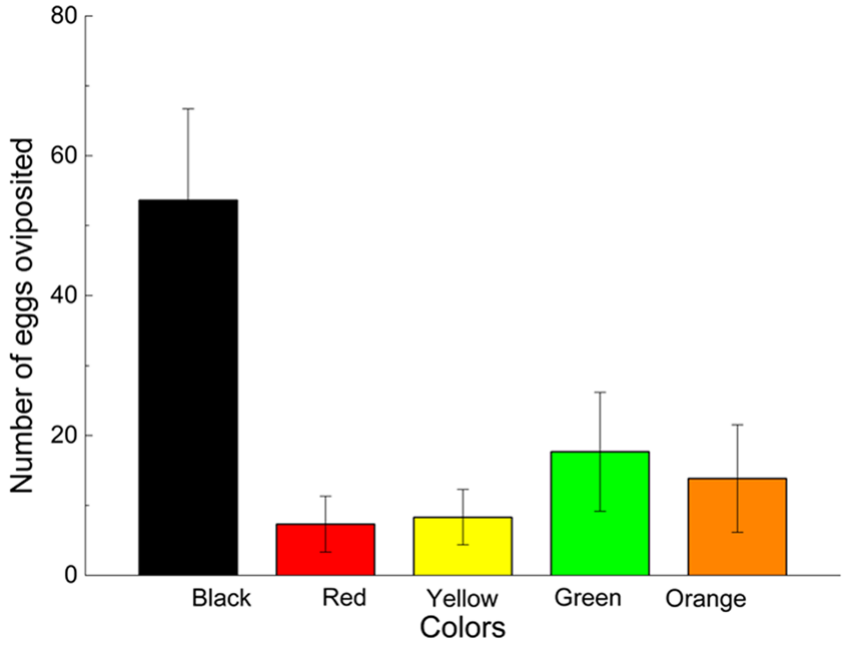
Number of eggs per female per day oviposited on different-colored substrates in the multiple-choice experiments in the innate-preference experiment. The mean number of eggs laid per Petri dish on each color of substrate using the chi-squared goodness of fit test (*X*^2^ = 74.693, *df* = 4, *P* < 0.001).

In the multiple-choice test, the parasitic beetles preferentially chose the black substrate for oviposition, followed by the green, orange, red and yellow substrates: The mean number of eggs laid per Petri dish per day on the black, green, orange, yellow and red substrates was 53.63 ± 13.07 (mean ± S.E., the same below), 17.68 ± 8.50, 13.84 ± 7.70, 8.32 ± 3.97, and 7.32 ± 4.00, respectively. The number of eggs differed significantly among the different substrate colors (*X*^2^ = 74.693, *df* = 4, *P* < 0.001); with more eggs were laid on the black substrate than on the green, orange, yellow or red substrates (Fig. 1).

**Figure 2:**
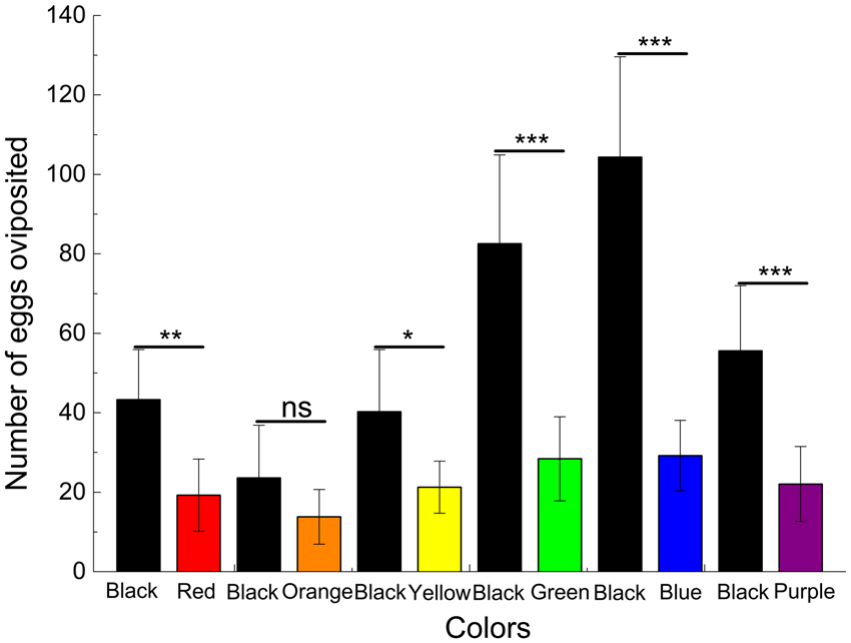
Number of eggs per female per day oviposited on different-colored substrates in the dual-choice experiments in the innate preference experiment. ns: *P* > 0.05, *P < 0.05, **P < 0.01, ***P < 0.001 by chi-squared tests at α = 0.05 level; all *df* = 1.

In the dual-choice experiments, *D. helophoroides* females also showed a clear oviposition preference for black substrate over the other substrate colors tested (Fig. 2). When the black substrate was tested against the red, yellow, green, blue and purple substrates, females laid more eggs on the black substrate than the other substrates (black vs. red: *X*^2^ = 9.290, *df* = 1, *P* = 0.002; black vs. yellow: *X*^2^ = 5.918, *df* = 1, *P* = 0.015; black vs. green: *X*^2^ = 27.252, *df* = 1, *P* < 0.001; black vs. blue: *X*^2^ = 42.293, *df* = 1, *P* < 0.001; black vs. purple: *X*^2^ = 14.767, *df* = 1, *P* < 0.001). The respective mean number of eggs on the black and red substrates was 43.30 ± 12.58 and 19.25 ± 9.09; on the black and yellow substrates was 40.25 ± 15.64 and 21.25 ± 6.55; on the black and green substrates was 82.55 ± 22.38 and 28.40 ± 10.58; on the black and blue substrates was 104.35 ± 25.26 and 29.20 ± 8.86; and on the black and purple substrates was 55.55 ± 16.43 and 22.05 ± 9.45. However, when the insects were tested using black against orange, the beetles did not display any significant substrate preference, although they laid more eggs on the black substrate (*X*^2^ = 2.63, *df* = 1, *P* = 0.105); the mean number of eggs laid per Petri dish per day on the black and orange substrates was 23.60 ± 13.24 and 13.80 ± 6.89, respectively.

### Associative learning

**Figure 3:**
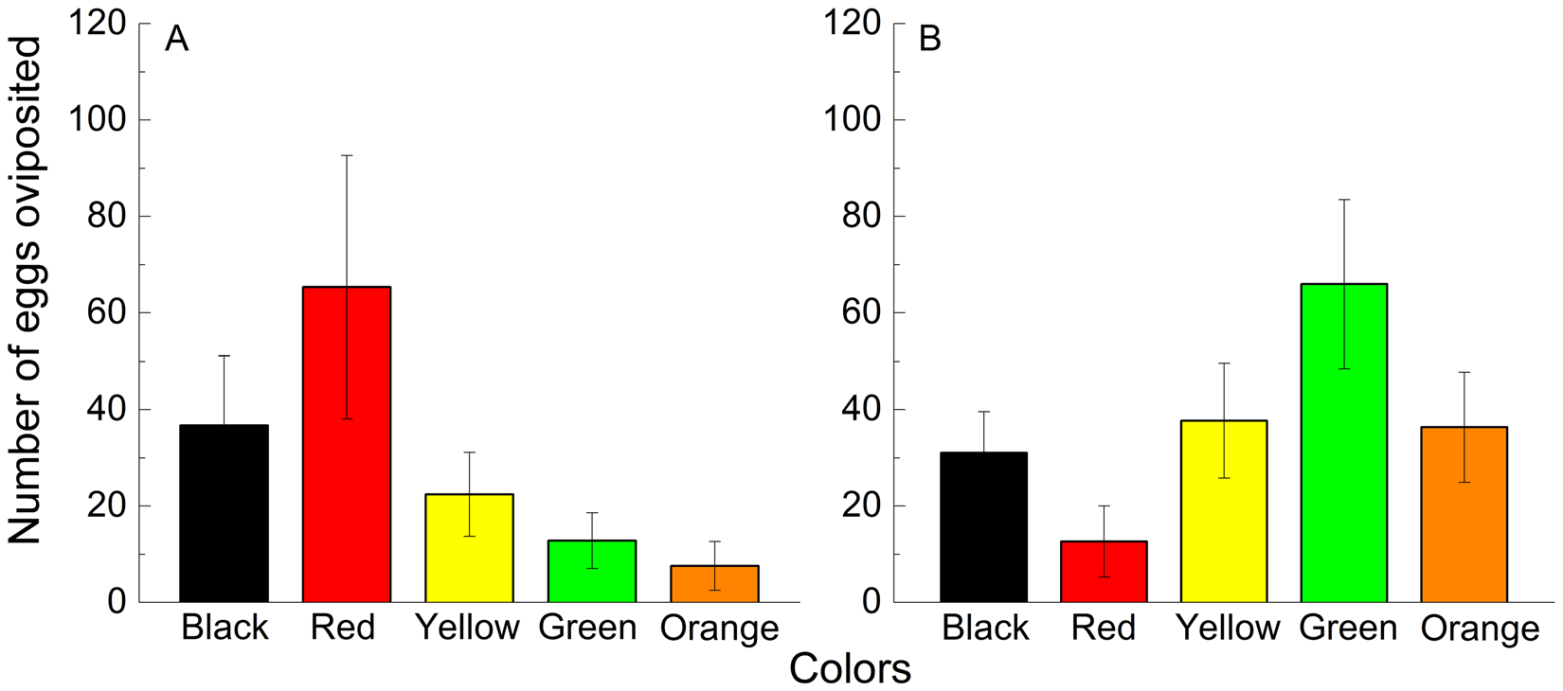
Number of eggs per female per day on substrates of different colors in the multiple-choice tests of associative learning. (**A**) Training on the red substrate, (**B)** training on the green substrate. The mean number of eggs laid per Petri dish on each color of substrate on the test session using the chi-squared goodness of fit test, when the test insects were trained on the red (*X*^2^ = 74.611, *df* = 4, *P* < 0.001) and green (*X*^2^ = 39.533, *df* = 4, *P* < 0.001) substrate.

In both the multiple- and dual-choice experiments, some eggs were laid under the wood and on filter paper during the 10-day training period when the beetles were trained on the different colored substrates. In the multiple-choice experiments, when training occurred on the red substrate (Fig. 3A), the females oviposited more eggs on red substrates than on the substrates of other colors in the test section (*X*^2^ = 74.611, *df* = 4, *P* < 0.001), and the mean number of eggs laid per Petri dish per day on the black, red, yellow, green and orange substrates was 36.67 ± 14.45, 65.33 ± 27.27, 22.39 ± 8.72, 12.78 ± 5.79, and 7.56 ± 5.06, respectively. When the insects were trained on the green substrates, significant differences were found in the numbers of eggs laid on substrates of different colors (*X*^2^ = 39.533, *df* = 4, *P* < 0.001) in the test session (Fig. 3B); the mean number of eggs laid per Petri dish per day on the black, red, yellow, green and orange substrates was 31.05 ± 8.54, 12.64 ± 7.37, 37.68 ± 11.91, 65.95 ± 17.54, and 36.32 ± 11.41, respectively.

**Figure 4:**
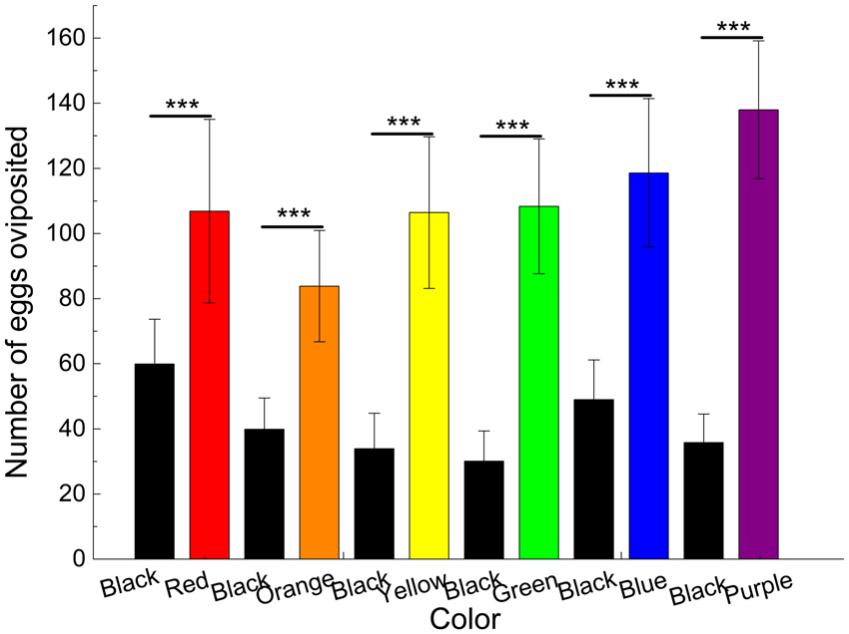
Number of eggs per female per day on substrates of different colors in the dual-choice tests of associative learning. *P < 0.05, **P < 0.01, ***P < 0.001 by chi-square tests at α = 0.05 level; all *df* = 1.

In the dual-choice experiments (Fig. 4), after the parasitic beetles were trained in the presence of black (without wood) and red, orange, yellow, green, blue and purple (with wood) substrates, the females laid significantly more eggs on the red, orange, yellow, green, blue and purple substrates than on the black substrate in the test (13.23 ≤ *X*^2^ ≤ 59.79, *df* = 1, all *P* < 0.001). The mean number of eggs on the black and red substrates, 59.93 ± 13.75 and 106.87 ± 28.17; on the black and orange substrates, 39.90 ± 9.56 and 83.83 ± 17.08; on the black and yellow substrates, 33.93 ± 10.89 and 106.43 ± 23.30; on the black and green substrates, 30.10 ± 9.24 and 108.33 ± 20.68; on the black and blue substrates, 48.97 ± 12.18 and 118.60 ± 22.80; and on the black and purple substrates, 35.80 ± 8.77 and 138.00 ± 21.14, respectively.

## Discussion

The results show that parasitic *D. helophoroides* females have an innate preference for ovipositing on black substrates. The preference for this color was changed by training on other colors with artificial oviposition sites, as *D. helophoroides* females laid more eggs on substrates of the color used in training than on black substrates. For example, the parasitic beetles laid eggs predominantly on the black substrate in the innate preference test, but when the insects were trained using a black substrate without wood and another color with wood, the parasitic beetles chose the conditioned substrate for oviposition, and the number of eggs laid on that substrate was significantly greater than that laid on the black substrate in the test session.

Two hymenopteran parasitoids were previously shown to prefer yellow over other colors^7^. Hymenopterans exhibit maximum spectral sensitivity to radiation in the range of UV, blue, green/yellow, and yellow, as surfaces emit radiation in the range of the visible spectrum similar to that of the young foliage of plants; accordingly, these parasitoids and some phytophagous insects use color cues (e.g., yellow) to locate appropriate hosts and host microhabitats^7,22^. However, in the multi-choice experiments, we found that parasitic *D. helophoroides* females have an innate preference for ovipositing on black substrates. The very low reflectance of the substrates preferred by these parasitic beetles could also represent the use of a cue related to the habitat of the host (i.e., the coloration of bark beneath the larval frass). In the current study, the reflectance spectrum range of the black substrates might be more similar than those of the other substrates to the spectrum reflected by tree bark. The parasitic beetle *D. helophoroides* is the predominant species among the natural enemies of the wood-boring tree insects, and it shows outstanding effectiveness in controlling large- and medium-bodied longhorn beetles, such as *A. glabripennis*, *Apriona germari*, and *M. alternatus*^13,14^. In previous studies on many forest pests, trunk pests particularly clearly prefer black to other colors in the field. For example, Campbell *et al*.^23^ suggested that the wood-boring beetle and the ambrosia beetle showed greater preferences for black traps than for white traps, and the female European woodwasp *Sirex noctilio* was trapped more frequently by a black flight intercept trap than by a clear jar trap^24^. In addition, many longhorn beetle species preferentially feed on plants that are suitable host plants for their larvae^11^. Thus, *D. helophoroides*, as a parasitic beetle, may depend on visual cues from damaged plants when searching the host site for a suitable egg-laying location. Therefore, our findings suggest that *D. helophoroides* may use visual cues to find appropriate oviposition sites during the initial stages of their search for and selection of host habitats.

In the dual-choice experiments, we aimed to determine the ability of these beetles to distinguish the color substrates at appropriate oviposition sites locations and expected that the beetles would be able to identify special colors. However, these beetles still showed an oviposition preference for black substrates. The parasitic wasps prefer yellow to the other colors because they have maximum spectral sensitivity to radiation in the range of UV, blue, green/yellow, and yellow (see above). However, some species of insect show a preference for black. For example, in the color trap experiments with the old house borer (*Hylotrupes bajulus*), the results showed that black colored traps were more effective than brown, yellow, red, grey, blue, white, and green traps^25^. The mosquitoes *Toxorhynchites moctezuma* and *Tx. amboinensis* also oviposited preferentially into black containers rather than white, red, yellow, green or blue containers^26^. For these species that show a black preference for oviposition or orientation, future surveys of the maximum spectral sensitivity range will be necessary. However, our results form dual-choice tests are consistent with the results of multi-choice tests, which further demonstrate that the beetles prefer black to the tested substrates of colors for oviposition.

Orange and yellow both belong to the approximate yellow region of the color spectrum. Although the female beetles in this experiment laid significantly more eggs on the black substrate than on the orange substrate, there was no significant difference between the orange and black substrates. However, females laid significantly fewer eggs on the yellow substrate than on the black one, indicating that the female beetles recognize slight differences in wavelength and intensity. However, yellow is reported to be a typical foliage stimulus for aphids, psyllid species and others, and numerous insect species show a preference towards yellow^27–29^. The response to color depends on the consumption of certain parts of host by a given insect species^30^. For insects that specialize in feeding on leaves and flowers, yellow may be more important than black or green. By contrast, for *D. helophoroides*, which parasitize the larvae of longicorn beetles, black and orange colors similar to those of bark and larval frass may be preferred over green and yellow. Further research should focus on enhancing our knowledge of host-finding behavior in the ability of *D. helophoroides* to recognize different intensities of yellow and black, especially because this beetle is potentially an excellent natural enemy of longhorn beetles. Furthermore, in Aquino *et al*.^7^ used sample sizes of 19–25 female adults, and found that two parasitoids continued to show a preference for yellow substrates after being trained on substrates of different colors, whereas Lucchetta *et al*.,^31^ used sample sizes of 20–168 *Venturia canescens* females and trained them to associate an orange-colored stimulus with a food reward. In our research, a sample size of 20 replicates was used, and the small number of sample replicates might have been the reason that the female beetles did not show a significant difference in color preference between black and orange substrates; however, Rosenberg (2010)^32^ provided an equation for calculating effect sizes for Chi-square tests and found that the effect size was 0.36 in the comparison between black with orange substrates, which indicates that if the sample size were to be increased, the mean number of eggs laid on the black substrate might be significantly greater than that on the orange substrate^33^. Moreover, we speculate that similarity of the orange color to the larval frass color was the reason for this finding.

To date, there have been no relevant studies regarding whether *D. helophoroides* has clear preferences for particular visual cues (color, shape, etc.) from its host plants or the frass of its host larvae^13,14^. Many studies have focused on determining whether the combination of visual and olfactory stimuli, compared to visual or olfactory stimuli alone, may enhance the accuracy with which insects discriminate hosts and host habitats. Indeed, many insects have been shown to respond to host plants and mates via synergistic visual, olfactory and tactile cues^34–37^. Moreover, in a previous study, Wei *et al*.^6^ showed that (*S*)-(*−*)-limonene from cerambycid larval frass was an important component of the kairomone attracting *D. helophoroides* adults in their search for larval *A. glabripennis*. A great deal of work has suggested that chemicals play a major role in the host-searching behavior of *D. helophoroides*. Therefore, the responses of *D. helophoroides* adults to the interaction between visual (particularly color) and olfactory cues from their hosts and host habitats will be the focus of our future research. The use of visual cues combined with chemical cues or cues of other modalities may provide better and more reliable information than any single modality for parasitic beetles in search of hosts and host habitats.

Because *D. helophoroides* is an important parasitoid of several species of longhorn beetles in China, these beetles are considered generalist or polyphagous parasitoids^13,14^. These parasitic beetles have a wide distribution in China^17^ and have different preferences for visual and chemical cues in different environmental contexts. For example, (*S*)-(*−*)-limonene was found to be an important component of the kairomone attracting *D. helophoroides* adults to parasitize larvae and pupae of *A. glabripennis* in Xi’an, China, while (*R*)-(*−*)-limonene and limonene were not important. However, *D. helophoroides* adults were attracted by all three of the abovementioned monoterpenes from parasitized *Apriona swainsoni* (Hope) in Qufu, Shandong, China. Wei *et al*. suggested that this difference in preference may have arisen because populations have different geographic origins, necessitating their adaptation to different ecological environments^6^. In agreement with this assertion, our experiments showed that 10 consecutive days of associative learning changed the choice patterns of the parasitic beetles in terms of the substrate colors on which they oviposited. Although no information has previously been reported about learning behavior in *D. helophoroides* adults, we found that their innate preference for black substrate was changed by associative learning with oviposition sites and successful oviposition on substrates of other colors as rewards. These parasitic beetles also have a long lifespan^38^, and Giunti *et al*.^3^ suggested that the habitat of a host may vary from one generation to the next, whereas it is typically constant within a generation. Therefore, many female parasitoids can learn to associate visual and olfactory information with oviposition sites. In our experiment, we found that *D. helophoroides* females are also able to learn visual associations; this capability enables them to rapidly adapt to different environments.

Non-host-foraging parasitoids encounter a trade-off between two mutually exclusive activities: searching for hosts and searching for food. Lucchetta *et al*.^31^ speculated that these parasitic insects can integrate information on three key parameters, i.e., the probability of finding food (food availability), metabolic reserves and host availability, to decide how they should allocate their time in searching for food and hosts. In the present study, a substitute host was used to artificially rear *D. helophoroides*, following established methods; however, over the long term, such rearing techniques could lead to recognition of the substitute host instead of the natural host. This study suggests that *D. helophoroides* females can use and learn visual cues from their experience in searching for oviposition locations. Therefore, if relevant cues of the target host were provided to adults to learn, the biological control abilities of these parasitic beetles for the target pests (food availability) could be enhanced.

Understanding the biology, ecology, and behavior of these parasitic beetles and their target pest species is essential for identifying and rearing high-quality natural enemies to ensure the success of biological control programs targeting specific pests^1^. Our results allow an improved understanding of the visual cues used by adult *D. helophoroides* during the search for oviposition sites. Giunti *et al*.^3^ indicated that visual cues, especially colors, often serve as valuable information for parasitic enemies at both close and long range when they are reliably associated with food or oviposition opportunities. Our experiment showed that adult *D. helophoroides* have the ability to learn using visual cues. *D. helophoroides* is the most effective natural enemy of large- and medium-bodied longhorn beetles^13,14^. Associative training with chemical and visual cues might be used to enhance the efficiency of biological control agents against a given target in mass-rearing protocols^3^.

## Materials and Methods

### Insects

Adult *D. helophoroides* individuals were provided by the Department of Entomology, Beijing Vocational College of Agriculture. The laboratory colony of *D. helophoroides* originated from parasitized larvae and pupae of *A. glabripennis*, and the *D. helophoroides* larvae were reared on a substitute host [*Thyestilla gebleri* (Fald.)]. The current study did not involve endangered or protected species. The adults used in the experiment were a second generation of beetles that had been reared in white plastic cages at 25 ± 1 °C, 50 ± 10% relative humidity and 1500–2000 lux under a photoperiod of 16 h light/8 h dark in an artificial climate chamber (RXZ-500D, Jiangnan Instrument, Ningbo, China). Two plastic centrifuge tubes (10 mm in diameter and 50 mm in length) were plugged with sponge balls to provide water, and *Tenebrio molitor* L. larvae were baked at 60 °C to serve as food for the adults. The water and diet were renewed every 5 days. Adult males and females were used in the behavioral tests; the sexes were distinguished by the end angle and the ratio of length to width of the anal plate under a dissecting microscope (Olympus SZ51, Tokyo, Japan) as described by Tang *et al*.^39^. All adults were used 60–90 days after emergence in our experiment, and all of the individuals were able to mate and oviposit normally. To reduce the effects of color on preimaginal and early adult life, we housed all of the adult beetles in a white environment (white plastic cages, white filter paper, white plastic centrifuge tubes and white wood). Therefore, the early adults had not experienced any color except white before their use in the bioassays.

### Experimental conditions

**Figure 5:**
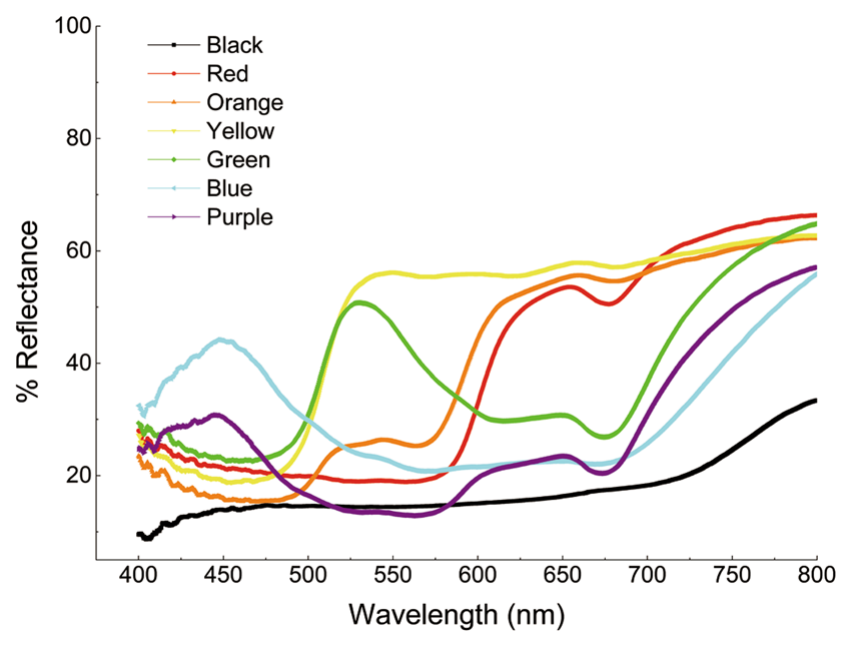
Reflectance spectra of the colored paperboard used in the experiment.

We conducted all of the experiments in an artificial climate chamber (RXZ-500D, Jiangnan Instrument) with white walls and a white ceiling. The chamber was illuminated with daylight fluorescent tubes (approximately 1500–2000 lux), and the experiments were conducted at 25 ± 1 °C and 50 ± 10% relative humidity under a photoperiod of 16 h L/8 h D. We applied a series of colors similar to those of the bark of plant branches and the frass of host larvae to photo printer paper (Deli3541; Deli Group Co., Ltd., Ningbo, China) using a printer (HP Color LaserJet Professional CP5225n; Hewlett-Packard Co., Palo Alto, CA, USA). A detailed description of the characteristics of the colors used in the tests is provided in Table 1. To accurately determine the colors of the substrates used in the bioassays, we measured the spectral reflectance of the colored paperboard using a spectroradiometer (USB Ocean Optics 2000+ , USA) equipped with a tungsten-halogen lamp light source (Fig. 5) following the procedure of Niu *et al*.^40^. An optic fiber (R-400-7 UV-VIS; Ocean Optic) connected to the spectrophotometer captured the reflected light from each substrate sample, and the percentage of reflected spectrum of each colored substrate was expressed relative to a white standard surface. The spectral readings were replicated three times for paperboard of each color in random order.

**Table 1:** Spectral characteristics used to quantify color.

Serial number	Color name	HEX	CMYK values	RGB values
Color 1	Red	#FF0000	0, 96, 95, 0	255, 0, 0
Color 2	Orange	#FFA500	0, 46, 91, 0	255, 165, 0
Color 3	Yellow	#FFFF00	10, 0, 83, 0	255, 255, 0
Color 4	Green	#00FF00	61, 0, 100, 0	0, 255, 0
Color 5	Blue	#0000FF	91, 75, 0, 0	0, 0, 255
Color 6	Purple	#800080	65, 100, 18, 0	128, 0, 128
Color 7	Black	#000000	93, 88, 89, 80	0, 0, 0

We used two types of glass Petri dishes as test chambers following Aquino *et al*.^7^ and based on the life habits of these beetles. To prevent the different treatments from influencing one another, we pasted white filter paper to the wall and bottom of each glass Petri dish. In our experiments, pieces of different-colored paperboard were pasted onto the filter paper, and a wood block with a concave trough (1.5 cm high × 1.5 cm wide and 1.5 cm long) was then placed on top of the filter paper to create a microhabitat for oviposition by the parasitic beetles. Three female and three male beetles were introduced into the experimental arena at the center of each glass Petri dish for behavioral testing.

### Innate preference

Multiple- and dual-choice experiments were conducted to establish whether the parasitic beetle exhibit innate color preferences and determine their ability of distinguish different color substrates in terms of their oviposition behavioral responses to substrates of different colors in closed arenas (i.e., the glass Petri dishes).

#### Multiple-choice experiment

**Figure 6:**
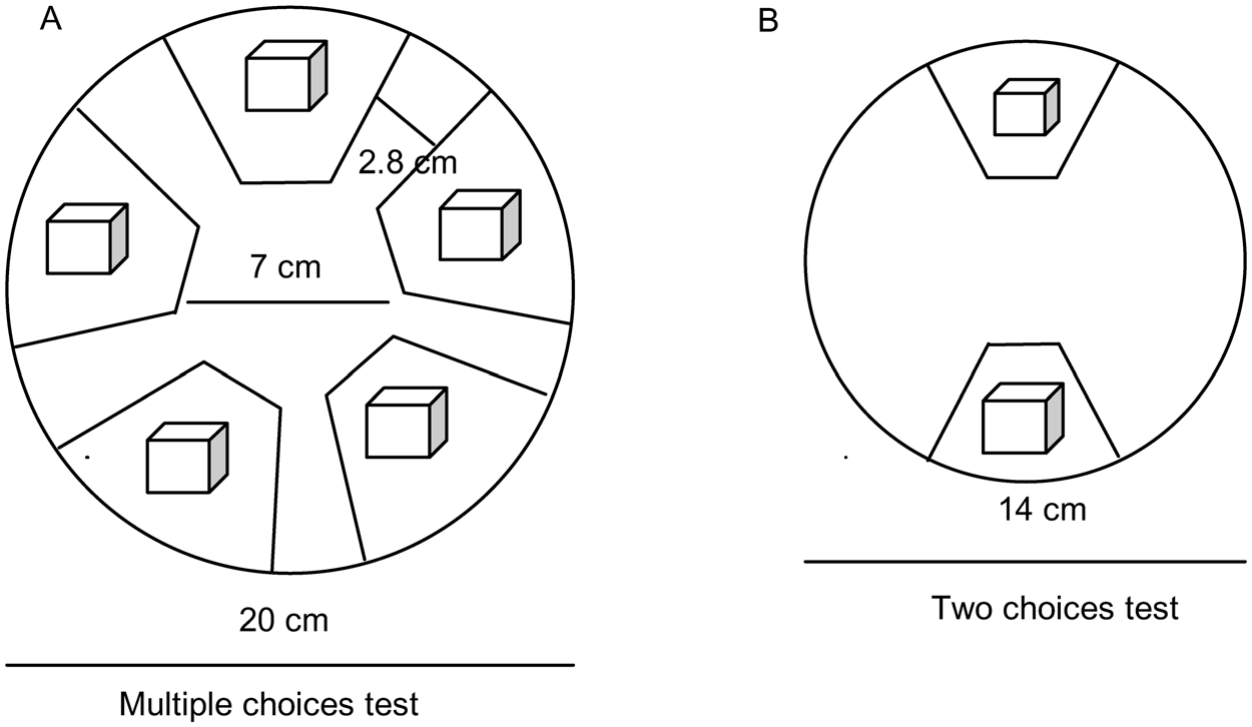
Schematic representations of the multiple- and dual-choice innate preference experiments. Top view of the arenas with diameters of (**A**) 20 cm and (**B**) 14 cm used in the multiple- and dual-choice experiments, with colored areas and a wood block with a concave trough in the center of each.

To determine whether the beetle uses visual cues (color substrate) to locate oviposition sites, multiple-choice experiments were conducted in closed arrears made with the Petri dish. The glass Petri dish arenas (20 cm diameter, 2.5 cm high) were divided into five trapezoidal colored areas (black, red, yellow, green and orange) of 26.5 cm^2^ each. Wood blocks, each one featuring a carved trough, were placed in the center of each colored area (Fig. 6A) to provide a concealed microhabitat for adults to shelter and oviposit. A free space of 2.8 cm in width separated the five trapezoidal areas of different colors, which were randomly positioned in the Petri dishes. The glass Petri dishes were then placed in an artificial climate chamber under the previously described test conditions.

Three females and three males of *D. helophoroides* were successively introduced to the center of each arena. The adults remained in the glass Petri dishes for a period of 24 h (i.e., from 8:00 the first day to 8:00 the next day) to allow oviposition to occur. Female *D. helophoroides* typically laid clusters of eggs on the colored paperboard beneath the wood blocks. After 24 h, the number of eggs on each color of substrate was counted under a dissecting microscope (Olympus SZ51, Tokyo, Japan). Nineteen groups (19 replicates × 3 pairs of adults) were tested.

#### Dual-choice experiment

The aim of the dual-choice experiment was to test the ability of *D. helophoroides* to distinguish special color cues in the comparison black with different color (red, orange, yellow, green, blue and purple) substrate. Two trapezoidal colored areas of approximately 12.5 cm^2^ each were placed opposite each other in an experimental arena made of a glass Petri dish (14 cm diameter and 2.5 cm high). A wood block with a trough was placed in the center of each colored area (Fig. 6B), after which the Petri dishes were placed in an artificial climate chamber under the previously described test conditions. Three females and three males were introduced to the center of each arena, and the adults were maintained in the Petri dish for a period of 24 h to allow oviposition to occur. Each colored substrate was evaluated for the presence of eggs after 24 h. The number of eggs was counted under a dissecting microscope. A set of experiments was conducted: (i) black vs. red, (ii) black vs. orange, (iii) black vs. yellow, (iv) black vs. green, (v) black vs. blue, and (vi) black vs. purple. Twenty groups (20 replicates × 3 pairs of adults) were tested in every experiment.

### Associative learning

To test whether the parasitic beetles could learn to associate the color of the substrate with the presence of a simulated oviposition microhabitat, we conducted two sets of experiments after a training period: (i) one offering multiple-choices to the parasitic beetles, and (ii) another offering dual-choices.

#### Multiple-choices assay after associative learning

**Figure 7:**
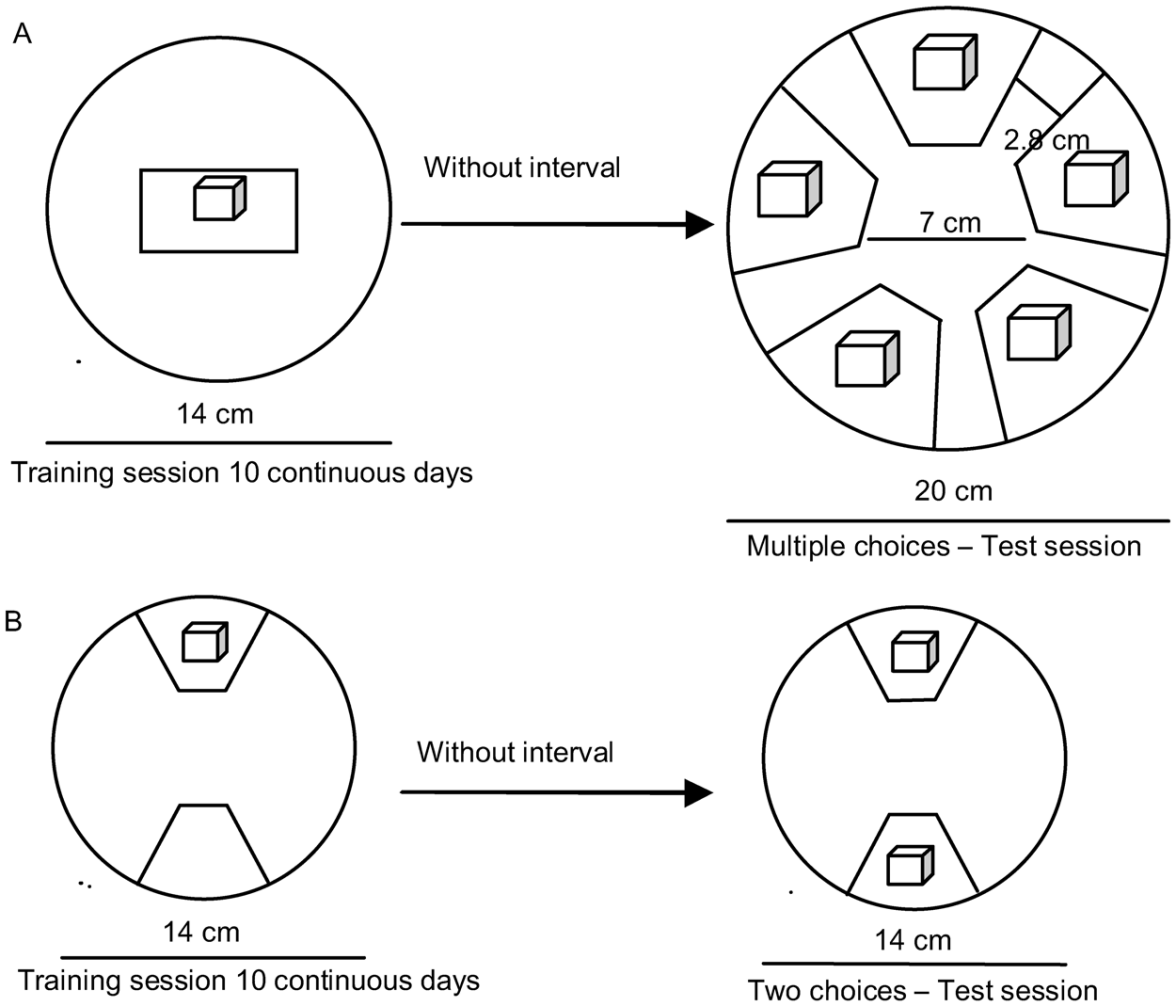
Schematic representations of the multiple- and dual-choice associative learning experiments. (**A**) Arenas (14 cm or 20 cm in diameter) used in the training and test sessions in the multiple-choice experiment. (**B**) Arenas (14 cm in diameter) used in the training and test sessions in the dual-choice experiment.

In this experiment, the parasitic beetles were trained in a glass Petri dish (14.0 cm diameter, 2.5 cm high) fully lined with white filter paper. Rectangular pieces of red and green paperboard (24 cm^2^) were placed in the center of the filter paper arena, and wood blocks with concave troughs were placed in the center of the colored substrate (Fig. 7A). Three pairs of adults were introduced into one side of the arena, and the training sessions were repeated for 10 consecutive days. After training, the adults were transferred to the center of a multiple-choice test arena (as described above, Fig. 7A), and the number of eggs was counted under a dissecting microscope after 24 h. Twenty groups (20 replicates × 3 pairs of adults) were used in every experiment.

#### Dual-choice assay after associative learning

The training and testing sessions for this experiment were conducted in arenas 14.0 cm in diameter. The arenas contained two trapezoidal areas of 12.5 cm^2^ each (Fig. 7B); these two colored areas were placed 7.0 cm apart at opposite sides of the arena. The substrates of different colors were placed randomly in the arenas to avoid possible position effects. In the training session, three female and three male adults were introduced into the center of the glass Petri dishes and remained in these dishes for 10 consecutive days. Then, in the testing session, the adults were moved to the test dishes for a period of 24 h, and the number of eggs on each substrate was counted under a dissecting microscope. A series of experiments were conducted in the following sequence. First, training was conducted on a black substrate without wood and on other substrates (including red, orange, yellow, green, blue and purple substrates) with wood; then, testing was performed on the black and other-color substrates, both with wood: (i) black vs. red, (ii) black vs. orange, (iii) black vs. yellow, (iv) black vs. green, (v) black vs. blue, and (vi) black vs. purple. Twenty groups (20 replicates × 3 pair adults) were tested in each experiment.

### Statistical analyses

To assess the influence of substrate color on oviposition, we compared the mean number of eggs laid per Petri dish on each color of substrate using the chi-squared goodness of fit test for the circular data from the multiple-choice experiment^41^. In each set of dual-choice experiments, the mean numbers of eggs laid per Petri dish on the different substrate colors were analyzed by an observed versus expected chi-squared test to determine whether the choice of different colors was significantly different from chance (i.e., all substrate colors having the same probability of being chosen for oviposition). All statistical analyses were performed using IBM SPSS Statistics 21.00 for Windows (IBM SPSS Inc., Boston, MA, USA)^42^.
